# Molecular Signaling Mechanisms for the Antidepressant Effects of NLX-101, a Selective Cortical 5-HT_1A_ Receptor Biased Agonist

**DOI:** 10.3390/ph15030337

**Published:** 2022-03-10

**Authors:** Sharon Cabanu, Fuencisla Pilar-Cuéllar, Paula Zubakina, Eva Florensa-Zanuy, Júlia Senserrich, Adrian Newman-Tancredi, Albert Adell

**Affiliations:** 1 Instituto de Biomedicina y Biotecnología de Cantabria (IBBTEC), Consejo Superior de Investigaciones Científicas (CSIC)-Universidad de Cantabria, 39011 Santander, Spain; scabanu@gmail.com (S.C.); pilarmf@unican.es (F.P.-C.); paula.zubakina@gmail.com (P.Z.); evaflozan@gmail.com (E.F.-Z.); julia.senserrich@unican.es (J.S.); 2 Departamento de Fisiología y Farmacología, Facultad de Medicina, Universidad de Cantabria, 39011 Santander, Spain; 3 Centro de Investigación Biomédica en Red de Salud Mental (CIBERSAM), Instituto de Salud Carlos III, 28029 Madrid, Spain; 4 Neurolixis SAS, 2 Rue Georges Charpak, 81100 Castres, France; anewmantancredi@neurolixis.com

**Keywords:** mTOR, ERK1/2, GluA1, p11, BDNF, Akt, dopamine, glutamate, medial prefrontal cortex

## Abstract

Depression is the most prevalent of the mental illnesses and serotonin (5-hydroxytryptamine, 5-HT) is considered to be the major neurotransmitter involved in its etiology and treatment. In this context, 5-HT_1A_ receptors have attracted interest as targets for therapeutic intervention. Notably the activation of presynaptic 5-HT_1A_ autoreceptors delays antidepressant effects whereas the stimulation of postsynaptic 5-HT_1A_ heteroreceptors is needed for an antidepressant action. NLX-101 (also known as F15599) is a selective biased agonist which exhibits preferred activation of cortical over brain stem 5-HT_1A_ receptors. Here, we used behavioral, neurochemical and molecular methods to examine the antidepressant-like effects in rats of a single dose of NLX-101 (0.16 mg/kg, i.p.). NLX-101 reduced immobility in the forced swim test when measured 30 min but not 24 h after drug administration. NLX-101 increased extracellular concentrations of glutamate and dopamine in the medial prefrontal cortex, but no changes were detected in the efflux of noradrenaline or 5-HT. NLX-101 also produced an increase in the activation of pmTOR, pERK1/2 and pAkt, and the expression of PSD95 and GluA1, which may contribute to its rapid antidepressant action.

## 1. Introduction

Depression is a common mental disorder that affects approximately 300 million people worldwide. Further, its incidence has been growing since the COVID-19 pandemic outbreak, which poses an enormous challenge for mental health care. Although many current antidepressant drugs based on monoamine reuptake or monoamine oxidase inhibition were formulated as early as the 1950s–1960s, the efficacy of such therapies has not improved much since that time, and only showing amelioration in their diminished adverse effects profile. Moreover, even though the antidepressants elicit some therapeutic efficacy, they need to be taken for weeks or months before any meaningful clinical improvement emerges. More serious is the fact that approximately 30% of the patients have inadequate responses or no response at all to treatment [1,2,3]. In this context, the observation that ketamine, a widely used anesthetic drug, exerts a rapid antidepressant action within an hour of administration has been a breakthrough for the treatment of mood disorders [4,5]. Although some progress has been made in understanding the mechanism of action of ketamine, there are still aspects that need further investigation. For instance, recent preclinical work has shown that the serotonin (5-hydroxytryptamine, 5-HT) system in the brain is involved in the sustained antidepressant action of ketamine [6,7,8]. In this regard, it has been reported that the selective stimulation of 5-HT_1A_ receptors in the medial prefrontal cortex (mPFC) may be required for the antidepressant effects of ketamine [9], which agrees with previous work showing that the activation of postsynaptic 5-HT_1A_ receptors exerted rapid antidepressant effects [10,11].

Recent preclinical studies have demonstrated that the administration of the highly selective 5-HT_1A_ receptor biased agonist, NLX-101 (also known as F15599), results in a rapid antidepressant-like response, both in naïve animals [12,13] and rodent models of depression [14,15], presumably through the activation of cortical 5-HT_1A_ receptors. Indeed, direct cortical microinjection of NLX-101 reduced immobility in the forced swim test, an effect that was blocked by the selective 5-HT_1A_ receptor antagonist, WAY-100635 [16]. Furthermore, the administration of NLX-101 increases the dopamine efflux in the mPFC and decreases 5-HT in the hippocampus [17] although the contribution of these changes to the antidepressant response remains to be clarified. Remarkably, the anti-anhedonic effect of repeated administration of NLX-101 in the sucrose intake test was still present four weeks after cessation of treatment [14], suggesting that a sustained modification occurred, possibly through the activation of intracellular mechanisms promoting neuroplasticity [18]. In this regard, it has been observed that NLX-101 elicited a rapid stimulation of phosphorylated extracellular-regulated kinase 1/2 (pERK1/2) in the frontal cortex, an effect that was reversed by the coadministration of WAY-100635 [19]. In contrast, other 5-HT_1A_ receptor agonists induced increases and decreases in pERK1/2 in the frontal cortex and hippocampus, respectively, thus suggesting that behavioral effects of NLX-101 are possibly due through an action on cortical structures. However, a thorough study on the effects of NLX-101 on intracellular signaling pathways relevant to depression has not yet been performed.

To gain further insight into the procedures involved in the antidepressant-like effects of NLX-101, we have examined the time course changes in several synaptic proteins and intracellular signaling pathways. We have also studied the effects of the systemic administration of NLX-101 on the outflow of glutamate, noradrenaline, dopamine and 5-HT in the mPFC.

## 2. Results

The dose–response for the antidepressant-like effects of NLX-101 in several behavioral tests relevant to depression has been widely studied in previous work [12,13,14]. For this reason, we have chosen an optimal dose of the compound (i.e., 0.16 mg/kg, i.p.), near to the ED_50_ of the drug in the forced swim test (0.12 mg/kg) [12], to carry out our studies in Sprague-Dawley rats.

### 2.1. Behavioral Effects of NLX-101

**Figure 1:**
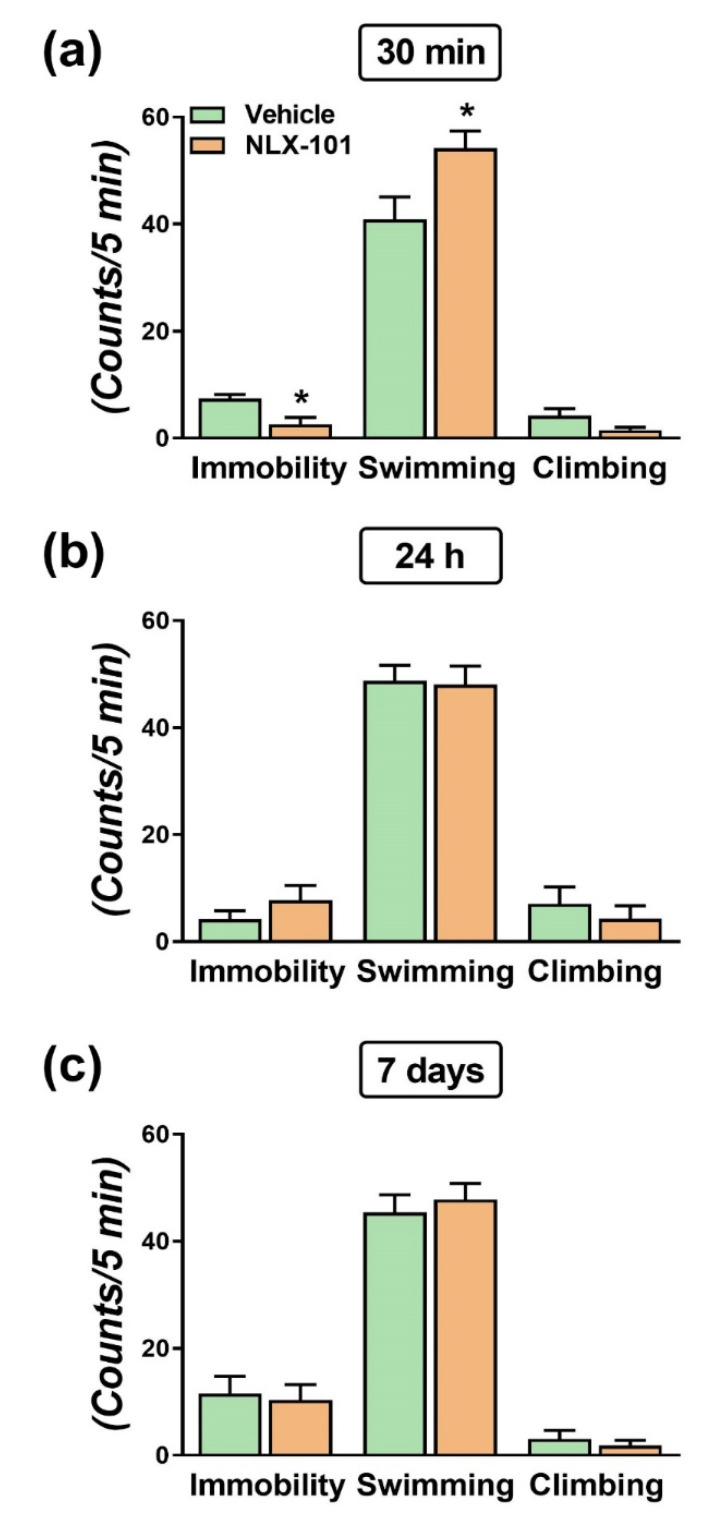
Antidepressant-like action of NLX-101 in the forced swim test (FST) conducted 30 min (**a**), 24 h (**b**) and 7 days (**c**) after the intraperitoneal injection of 0.16 mg/kg of NLX-101. Results are expressed as mean ± SEM of *n* = 5–6 rats/group, * *p* < 0.05, two-tailed Student’s *t*-test.

**Figure 2:**
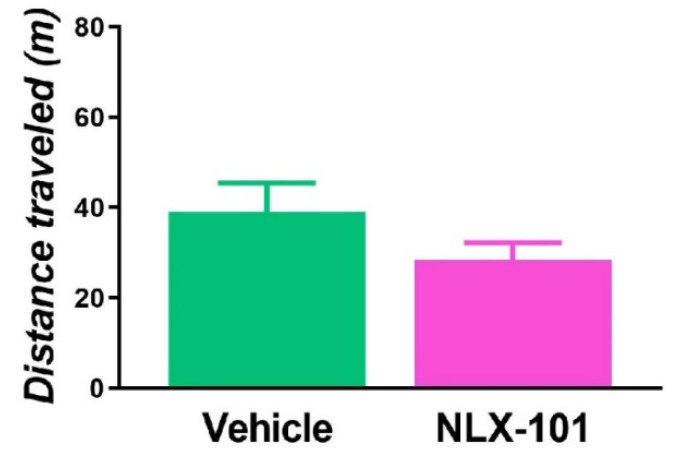
Behavioral response in the open field test (OFT). Locomotor activity after the administration of 0.16 mg/kg of NLX-101 is expressed as distance traveled in meters during 10 min. Results are expressed as mean ± SEM of *n* = 5–6 rats per group.

As shown in Figure 1a, 30 min after a single injection of 0.16 mg/kg of NLX-101, immobility in the FST was significantly reduced (*t* = 3.186, *df* = 8; *p* < 0.02, two-tailed Student’s *t*-test). This was accompanied by an increase in swimming (*t* = 2.527, *df* = 8; *p* < 0.05, two-tailed Student’s *t*-test). However, these antidepressant-like effects were not observed 24 h and 7 days after NLX-101 administration (Figure 1b,c). The NLX-101-induced decrease in immobility did not result from an altered locomotor activity as observed in the open field test (OFT) (Figure 2).

### 2.2. Biochemical Effects of NLX-101

**Figure 3:**
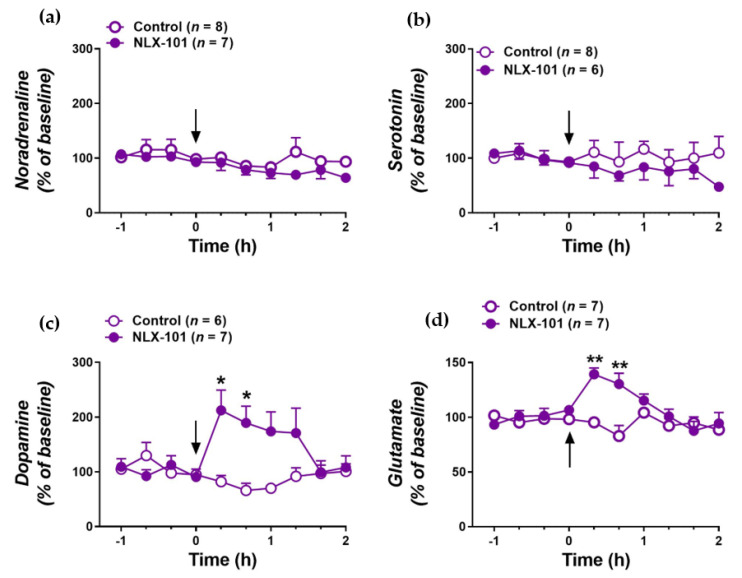
Effects of the administration of 0.16 mg/kg of NLX-101 or vehicle (arrow) on the extracellular concentration of noradrenaline (**a**), serotonin (**b**), dopamine (**c**) and glutamate (**d**) in the mPFC. Data (mean ± SEM) are expressed as percentage changes in the four basal pretreatment values. Number of animals is indicated in parentheses. * *p* < 0.05 and ** *p* < 0.0005 different from the corresponding vehicle group, Tukey’s multiple comparison test following significant two-way repeated measures ANOVA.

Microdialysis experiments showed that the same dose of NLX-101 that induced antidepressant-like effects (0.16 mg/kg, i.p.) did not alter the extracellular concentrations of noradrenaline (Figure 3a; *F*_1,12_ = 2.777; *p* = 0.121) or 5-HT (Figure 3b; *F*_1,12_ = 1.263; *p* = 0.283) in the mPFC, but significantly increased those in dopamine (Figure 3c) and glutamate (Figure 3d). Repeated measures two-way ANOVA showed that the administration of 0.16 mg/kg of NLX-101 enhanced dialysate dopamine as measured by significant effects of treatment (*F*_1,11_ = 7.152, *p* < 0.03) and treatment x time interaction (*F*_9,99_ = 4.305; *p* < 0.0001). Similarly, NLX-101 increased dialysate glutamate as measured by significant effects of treatment (*F*_1,12_ = 7.351; *p* < 0.02), time (*F*_9,108_ = 4.311; *p* < 0.0001) and treatment x time interaction (*F*_9,108_ = 5.817; *p* < 0.00001).

### 2.3. Effects of NLX-101 on Prefrontal Protein Expression

**Figure 4:**
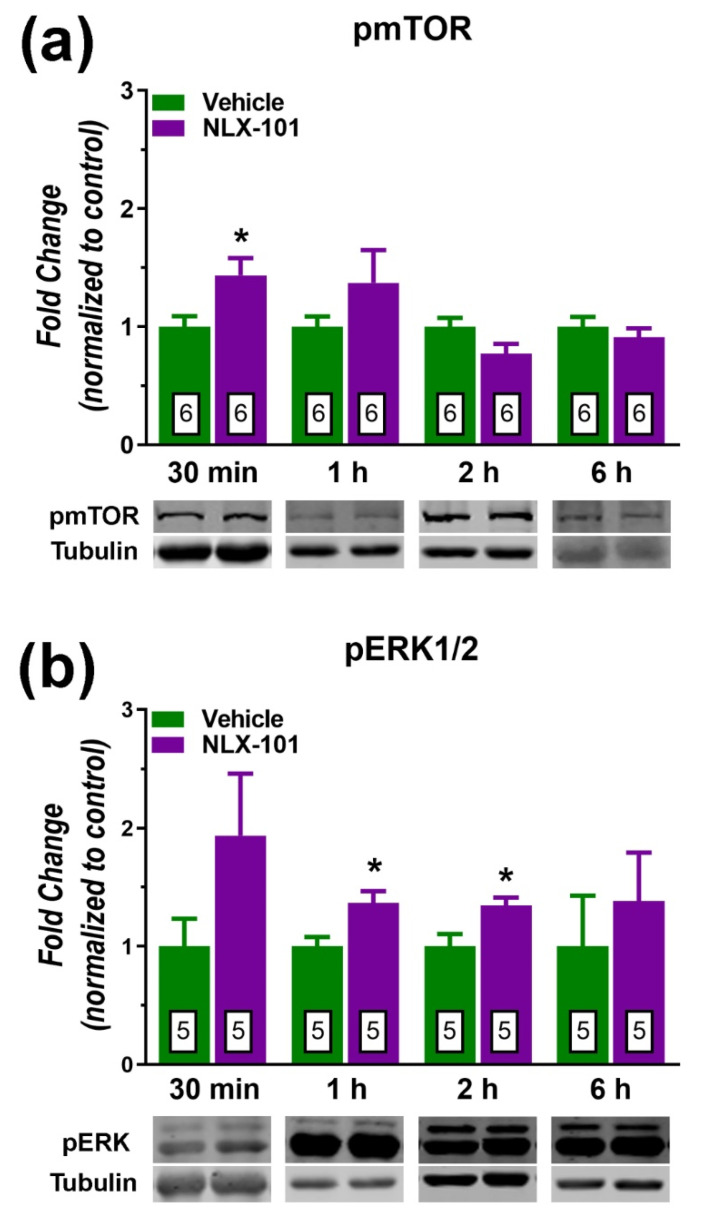
Effects of NLX-101 (0.16 mg/kg) and vehicle (Veh) on the concentration of pmTOR (**a**) and pERK1/2 (**b**) in the prefrontal cortex at 30 min, 1 h, 2 h and 6 h after its intraperitoneal administration. Results are expressed as mean ± SEM. Number of animals is indicated within the bars. * *p* < 0.05 compared with the corresponding vehicle group (two-tailed Student’s *t*-test).

**Figure 5:**
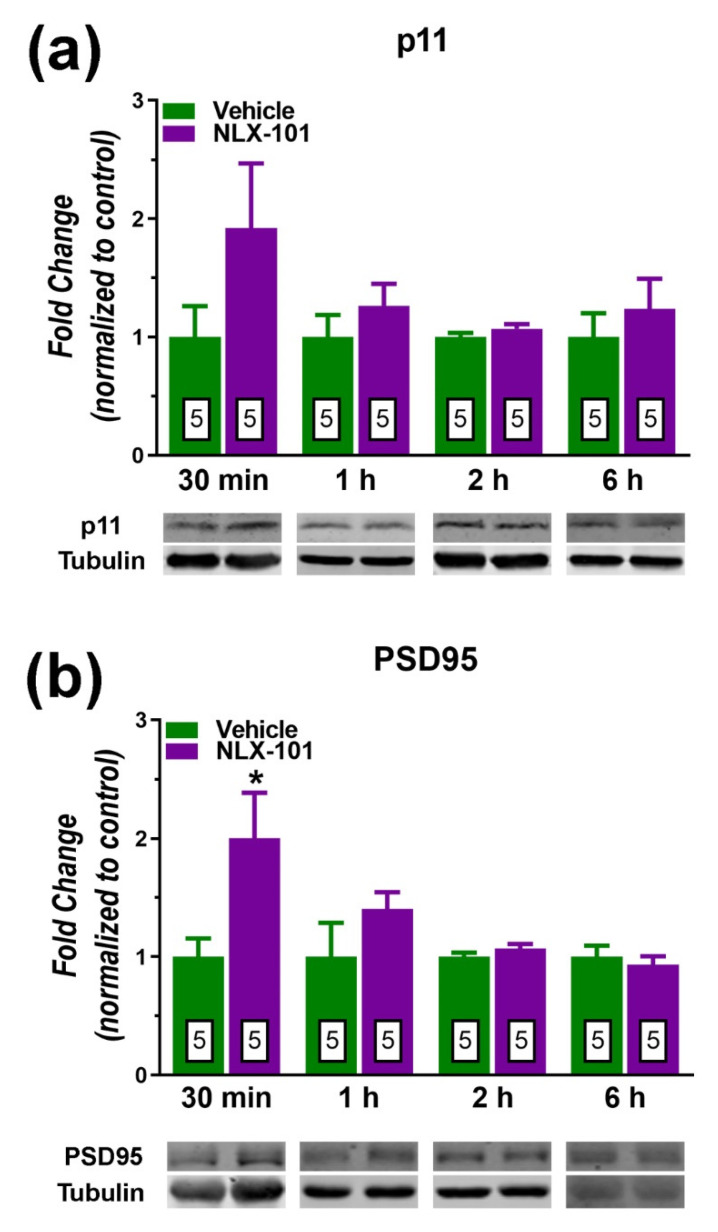
Effects of NLX-101 (0.16 mg/kg) and vehicle (Veh) on the concentration of postsynaptic proteins p11 (**a**) and PSD95 (**b**) in the prefrontal cortex at 30 min, 1 h, 2 h and 6 h after its intraperitoneal administration. Results are expressed as mean ± SEM. Number of animals is indicated within the bars. * *p* < 0.05 compared with the corresponding vehicle group (two-tailed Student’s *t*-test).

The synthesis of synaptic proteins in the PFC exhibited different time courses. Hence, as depicted in Figure 4a, the phospho–mammalian target of rapamycin (pmTOR) was the signaling protein that showed the fastest response (a 44% increase) 30 min after NLX-101 (*t* = 2.534, *df* = 10; *p* < 0.03, two-tailed Student’s *t*-test). No change in pmTOR was observed beyond this time point. As shown in Figure 4b, the level of pERK1/2 displayed significant increases at 1 h (*t* = 2.868, *df* = 8, *p* < 0.05, two-tailed Student’s *t*-test) and at 2 h (*t* = 2.773, *df* = 8, *p* < 0.05, two-tailed Student’s *t*-test). NLX-101 tended to increase the expression of pERK(1/2) at 30 min after its administration although this difference did not reach statistical significance (+94%, *t* = 1.634, *df* = 8, *p* > 0.05, two-tailed Student’s *t*-test). NLX-101 also produced a sizeable but not significant increase of the protein p11 (also known as S100A10) 30 later (+92%, *t* = 1.516, *df* = 8, *p* > 0.05, two-tailed Student’s *t*-test) as depicted in Figure 5a. Similarly, the postsynaptic density protein 95 (PSD95) increased 30 min after NLX-101 administration (*t* = 2.425, *df* = 8, *p* < 0.05, two-tailed Student’s *t*-test) as shown in Figure 5b.

**Figure 6:**
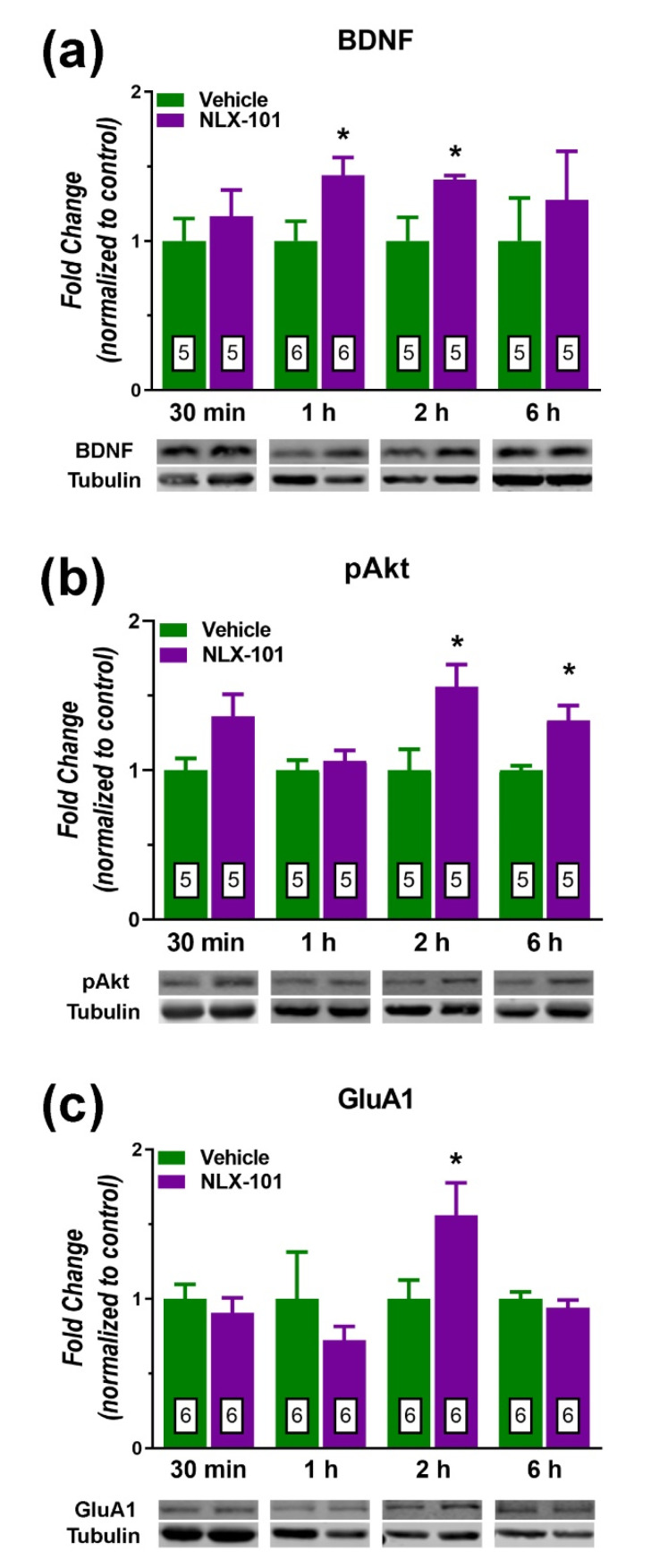
Effects of NLX-101 (0.16 mg/kg) and vehicle (Veh) on the concentration of BDNF (**a**), pAkt (**b**) and GluA1 (**c**) in the prefrontal cortex at 30 min, 1 h, 2 h and 6 h after its intraperitoneal administration. Results are expressed as mean ± SEM. Number of animals is indicated within the bars. * *p* < 0.05 compared with the corresponding vehicle group (two-tailed Student’s *t*-test).

**Figure 7:**
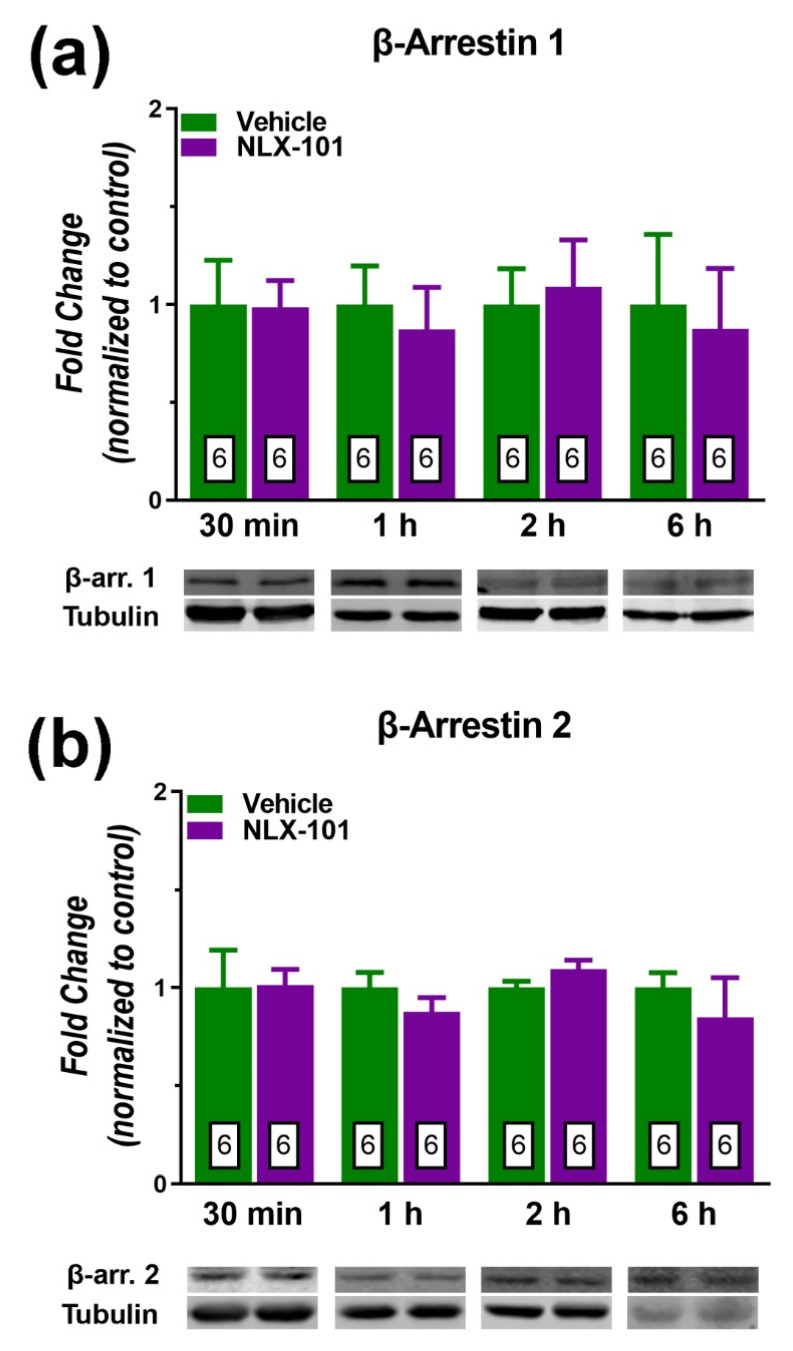
Effects of NLX-101 (0.16 mg/kg) and vehicle (Veh) on the concentration of β-arrestin 1 (**a**) and β-arrestin 2 (**b**) in the prefrontal cortex at 30 min, 1 h, 2 h and 6 h after its intraperitoneal administration. Results are expressed as mean ± SEM. Number of animals is indicated within the bars.

**Figure 8:**
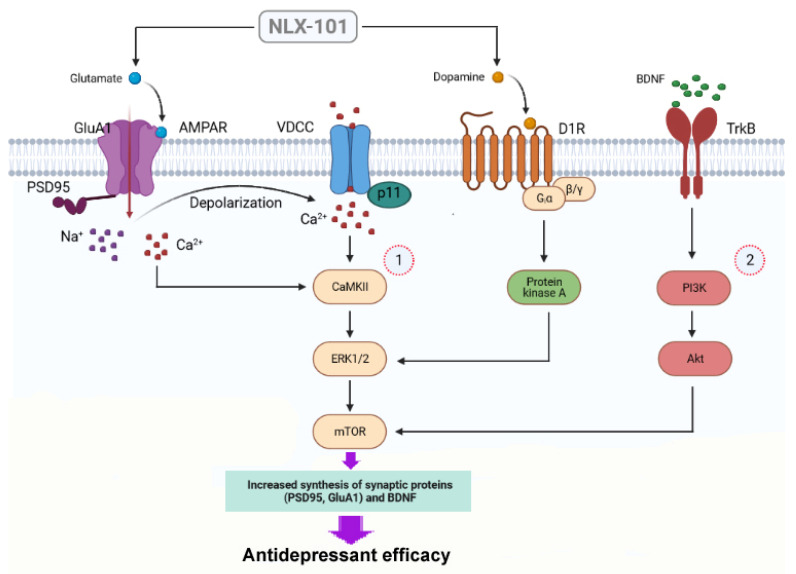
Scheme of the intracellular signaling pathways involved in the antidepressant-like effects of NLX-101. NLX-101 preferentially activates 5-HT_1A_ receptors expressed in GABA interneurons, thus reducing their activity and inducing a disinhibition of glutamatergic neurons with the subsequent release of glutamate and dopamine. Glutamate would evoke a rapid (1) stimulation of AMPA receptors (AMPAR) localized to the plasma membrane of pyramidal cells, which would result in a rapid intracellular activation of CaMKII that would eventually activate (phosphorylate) ERK1/2 and mTOR pathways, thus inducing a rapid synthesis of PSD95 and p11. The binding of dopamine to D1 receptors (D1R) can also contribute to the expression of ERK1/2 through activation of protein kinase A. A delayed antidepressant mechanism (2) would involve the mTOR-induced synthesis of BDNF that would bind to its receptor, TrkB, followed by downstream activation of Akt and synthesis of GluA1. Abbreviations: AMPA, α-amino-3-hydroxy-5-methyl-4-isoxazolepropionic acid; BDNF, brain-derived neurotrophic factor; CaMKII, Ca^2+^/calmodulin-dependent protein kinase II; D1, dopamine D1 receptor; ERK1/2, extracellular-regulated kinase 1/2; mTOR, mammalian target of rapamycin; PI3K, phosphatidylinositol-3 kinase; TrkB, tropomyosin receptor kinase B; VDCC, voltage-dependent calcium channel. Illustration created with BioRender.com, accessed on 26 January 2022.

In contrast, brain-derived neurotrophic factor (BDNF) (Figure 6a) and pAkt (Figure 6b) and GluA1 (Figure 6c) displayed a more delayed response to the administration of NLX-101. The expression of BDNF was significantly elevated at 1 h (*t* = 2.478, *df* = 8, *p* < 0.05) and 2 h after drug administration (*t* = 2.571, *df* = 8, *p* < 0.05), whereas the increase in the level of pAkt was significant at 2 h (*t* = 2.742, *df* = 8, *p* < 0.03) and 6 h (*t* = 3.273, *df* = 8, *p* < 0.02) after NLX-101. The expression of GluA1 subunit also increased significantly 2 h (+74%, *t* = 2.229, *df* = 10; *p* < 0.05, two-tailed Student’s *t*-test) after NLX-101 administration. NLX-101 did not alter the synthesis of β-arrestin 1 and β-arrestin 2 (Figure 7) at any of the tested time points. A schematic representation of the molecular signaling mechanisms involved in the antidepressant-like effects of NLX-101 is depicted in Figure 8.

## 3. Discussion

The principal findings of this study are that the cortical 5-HT_1A_ receptor biased agonist, NLX-101, reduced the immobility in the FST when measured 30 min after its administration. Systemic administration of NLX-101 increased the dialysate levels of glutamate and dopamine in the mPFC. In contrast, no changes were observed in the mPFC outflow of noradrenaline and 5-HT. NLX-101 also produced a rapid increase in the synthesis of pmTOR and PSD95, which may also contribute to its rapid antidepressant action.

### 3.1. Effects of NLX-101 on FST and Cortical Neurotransmitter Levels

The present work confirmed the antidepressant-like effects in rats administered a single dose (0.16 mg/kg i.p.) of NLX-101 on the FST [12,13]. The NLX-101-induced decrease in immobility behavior can be accounted for by an action on 5-HT_1A_ receptors since it was counteracted by the selective 5-HT_1A_ receptor antagonist WAY-100635 [12]. Although the change in immobility was notable when FST was performed 30 min after drug administration, this effect was not present 24 h and 7 days later, which was in line with previous work showing that the reduction in immobility for the FST only lasted for around 8 h [12]. Interestingly, the reduction in immobility was caused by an increase in swimming, which has been attributed to an activation of serotonin transmission [20,21]. However, our microdialysis results show that NLX-101 did not alter dialysate 5-HT and noradrenaline in the mPFC, suggesting a differential regulation for monoamine neurons by mPFC 5-HT_1A_ receptors. It is possible, though, that the increased swimming elicited by NLX-101 is caused by an increase in 5-HT in another brain region such as the nucleus accumbens [22] and further research is needed to determine the validity of this hypothesis. Similar to previous work [17], the dialysate level of dopamine in the mPFC is strongly influenced by NLX-101, a common response shared by antidepressants acting with different primary mechanisms [23]. It is postulated that the stimulation of dialysate dopamine is caused by a preferential activation of 5-HT_1A_ receptor in γ-aminobutyric acid (GABA) interneurons, which would disinhibit layer 5 pyramidal neurons projecting to the ventral tegmental area (VTA), thus subsequently activating mesocortical dopamine neurons [24]. The increase in firing rate of mPFC pyramidal neurons produced by NLX-101 [17] and the finding that the inhibition of GABA input to pyramidal neurons suppresses the pyramidal discharge rate increase evoked by the prototypical 5-HT_1A_ receptor agonist 8-OH-DPAT [25] would support this view. Moreover, in line with these results, it has been described that the stimulation of mPFC 5-HT_1A_ receptors increases phasic inputs onto dopaminergic neurons of the VTA [26] that project back to the mPFC [27]. Hence, increases in mPFC dopamine release may be involved in the improvement of mood, rewarding stimuli and cognitive dysfunction seen in depression [28,29,30,31]. As a matter of fact, optogenetic activation of VTA dopamine neurons reversed the anhedonic effects of a chronic stress model for depression [32].

Here we also described for the first time that the antidepressant dose of NLX-101 enhances the dialysate level of glutamate in the mPFC, an observation which is in line with the increased firing rate of mPFC pyramidal neurons seen previously [17]. Increased glutamate release in the mPFC has also been observed after a single administration of the rapid-acting antidepressant ketamine [33,34,35]. This rapid ketamine-induced glutamate burst stimulates α-amino-3-hydroxy-5-methyl-4-isoxazolepropionic acid (AMPA) receptors [7,36,37,38] and results in activity-dependent synapse formation in the mPFC [39,40,41,42]. It is thus conceivable that the glutamatergic effects of NLX-101 would induce similar neuroplasticity mechanisms. Overall, it is possible that the lack of effects on noradrenaline and 5-HT may be responsible for the shorter duration of the antidepressant-like effects of NLX-101 compared with ketamine, whereas the increases in cortical dopamine and glutamate mediate the rapid-acting antidepressant-like effects of the compounds.

### 3.2. Effects of NLX-101 on Intracellular Signaling Biomarkers

At a molecular level, NLX-101 rapidly influenced the cortical expression of pERK1/2, pmTOR and PSD95. The rapid increase in pmTOR expression is not maintained, thus suggesting that downstream mechanisms contribute to the behavioral effects of NLX-101. The preferential increase in the cortical phosphorylation of ERK1/2 agrees with a previous report [43] and, together with the rapid phosphorylation of mTOR, NLX-101 shares similar activity on these intracellular components as shown by ketamine [39,44]. The NLX-101-induced rapid increase in PSD95 would also contribute to these effects. p11 is a protein that can interact with multiple ion channels and G protein-coupled receptors [45]. The constitutive deletion of p11 in mice evokes behavioral changes relevant to a depressive-like phenotype in several well-established animal models [46,47]. In contrast, upregulation of p11 is associated with antidepressant effects [48]. Because p11 has an important function in the conveyance of transmembrane proteins [45], our results would support the view that a rapid increase in prefrontal p11—although the effect did not achieve statistical significance—could potentiate glutamatergic transmission, which in turn would contribute to synaptic plasticity [46]. In addition, our results showed, for the first time, that BDNF, pAkt and the AMPA receptor subunit GluA1 are increased by a single systemic injection of NLX-101, although with a time course which was slower compared with that of pmTOR or pERK1/2. Importantly, these effects are postulated to be a convergent mechanism underlying antidepressant action [49], and deficits in the expression of these proteins are associated with depression [50,51,52] and observed in stress-induced behaviors in animal models [53,54]. The expression of BDNF increased between 1 h and 2 h after drug administration, whereas the cortical level of pAkt only increased significantly beyond 2 h and GluA1 only 2 h after NLX-101 administration. These findings are at variance than those reported for ketamine, which increased BDNF [55] and pAkt [39] over a shorter period of time (within 30 min and 1 h). These differences may underlie the diverse onset and/or duration of antidepressant-like effects between ketamine and NLX-101, although direct head-to-head comparison studies would be necessary to confirm this interpretation. NLX-101 did not alter the expression of β-arrestins, which agrees with previous in vitro experiments showing that this compound more potently stimulated ERK1/2 phosphorylation than β-arrestin activation [43]. In summary, our present results suggest that neurochemical and molecular changes in the mPFC should participate in the antidepressant-like effects of NLX-101. Indeed, a predominant action in the mPFC is hypothesized to subserve the clinical efficacy of well-established rapid acting antidepressant entities such as ketamine [56,57], and our results suggest that this may also be the case for biased agonists such as NLX-101 that directly target cortical 5-HT_1A_ receptors in the mPFC.

## 4. Materials and Methods

### 4.1. Animals

Male Sprague–Dawley rats (Envigo RMS Spain S.L., Sant Feliu de Codines, Spain) weighing 240–280 were used in this study. The rats were group-housed and maintained in a controlled environment (12 h light/dark cycle, 22 ± 1 °C ambient temperature) with food and water ad libitum. All the experimental procedures were conducted in accordance with national (RD 53/2013) and European legislation (Directive 2010/63/EU, on the Protection of Animals Used for Scientific Purposes, 22 September 2010), and were approved by the Animal Care and Use Committee of the University of Cantabria and the Consejería de Medio Rural, Pesca y Alimentación (protocol code PI-08-17, approved on 7 March 2017). Rats were allowed one week of acclimatization before the start of experiments.

### 4.2. Drugs and Reagents

NLX-101 (also known as F15599), 3-chloro-4-fluorophenyl-[4-fluoro-4-[[(5-methylpyrimidin-2-ylmethyl)amino]methyl]piperidin-1-yl]methanone fumarate, was provided by Neurolixis and dissolved in distilled water for intraperitoneal (i.p.) administration. Noradrenaline, dopamine hydrochloride, serotonin hydrochloride (5-HT), glutamate and HPLC and other reagents were purchased from Sigma–Aldrich (Tres Cantos, Spain).

### 4.3. Forced Swim Test (FST)

Rats were handled daily for one week before the behavioral test. A modified version of the FST was carried out as previously described [20,58]. On day 1 (pretest), rats were placed in a clear plexiglass cylinder (46 cm height, 20 cm diameter) filled with water (24 ± 1 °C) to a height of 30 cm, for 15 min. Following this pretest, animals were returned to their home cages and dried under a lamp for 30 min. Twenty-four hours after the pretest, rats received NLX-101 (0.16 mg/kg, i.p.). Three tests of 30 min duration were conducted 30 min, 24 h and 7 days after drug administration. The test sessions were recorded (ANY-maze, Stoelting Europe, Dublin, Ireland) and immobility, climbing and swimming were scored by an experimenter blind to the treatment, as previously described [20].

### 4.4. Open Field Test (OFT)

To rule out any unspecific effects of NLX-101 that could interfere with FST behaviors, locomotor activity was evaluated using an open field arena (100 cm × 100 cm × 40 cm) and recorded for 10 min (ANY maze).

### 4.5. Microdialysis Procedure

Concentric dialysis probes with a 4 mm Cuprophan (pore size 10,000 Da) membrane length were homemade and implanted under pentobarbital anesthesia (60 mg/kg i.p.) in the mPFC (AP + 3.2 mm, L −0.6 mm, DV −6.0 mm; from bregma), according to Paxinos and Watson atlas [59]. Microdialysis experiments were carried out 48 h after surgery in freely moving rats by perfusing probes with artificial cerebrospinal fluid (aCSF: 147 mM NaCl, 3 mM KCl, 1.2 mM CaCl_2_, 1.2 mM MgCl_2_) at a continuous rate of 1.5 μL/min. Dialysate samples (30 μL every 20 min) were collected in microvials containing 5 μL of 10 mM perchloric acid. At the completion of experiments, rats were euthanized using an overdose of sodium pentobarbital and the brains were rapidly removed, frozen and stored at −80 °C until used. Brains were sectioned using a cryostat and probe placements were confirmed in histological sections stained with cresyl violet. Experimental data from animals that presented misplaced probes were discarded. Noradrenaline, dopamine, 5-HT and glutamate were determined using an Alexys Analyzer (Antec Scientific, Leiden, The Netherlands) with amperometric detection, following manufacturer’s methods. Briefly, monoamines were detected at +0.46 V using a 1.0 × 100 mm Acquity UPLC^®^ BEH C18, 1.7 µm column (Waters Cromatografía, S.A., Cerdanyola del Vallès, Spain) and glutamate was pre-column derivatized with *o*-phtalaldehyde and detected at +0.7 V using a 1.0 × 50 mm Acquity UPLC^®^ HSS T3, 1.8 µm column (Waters Cromatografía, S.A.).

### 4.6. Protein Extraction and Western Blotting

In a separate set of experiments, rats were administered NLX-101 (0.16 mg/kg, i.p.), and sacrificed 30 min, 1 h, 2 h and 6 h later. Their brains were removed, and the prefrontal cortices were dissected on ice and stored at −80 °C. Samples were homogenized (1:15) in homogenization buffer [10 mM HEPES (pH 7.9), 1.5 mM MgCl_2_, 100 mM KCl, 1 mM phenylmethylsulfonyl fluoride (PMSF), 0.2 mg/mL aprotinin, 10 μg/mL leupeptin, 10 μg/mL pepstatin A, 10 μg/mL antipain, 10 μg/mL chymostatin, 1 mM Na_3_VO_4_ and 1 mM NaF]. Homogenates were sonicated on ice-cold protein lysis buffer (homogenization buffer containing 1% Igepal^®^, 0.5% sodium deoxycholate, 0.1% SDS and 2.5 mM CHAPS) for 30 min. Homogenates were centrifuged for 10 min at 14,000 rpm and 4 °C, and the supernatants were collected.

For each sample, 55 μg of protein (in duplicate) was separated using SDS-PAGE gels (10% or 15% acrylamide), and then transferred to nitrocellulose membranes (Bio-Rad, Hercules, CA, USA). The blocking step was performed with 5% skimmed-milk for non-phosphorylated proteins or 3% skimmed-milk containing phosphatase inhibitors for an hour at room temperature (except for pAKT antibody, which was incubated with 5% skimmed milk), in Tris buffered saline (TBS-T: 50 mM Tris-HCl, pH 7.6, 150 mM NaCl and 0.05% Tween-20). Membranes were incubated overnight at 4 °C with the primary antibodies diluted in the corresponding blocking solutions.

The sources and dilution of primary antibodies used were: rabbit anti-pmTOR (1:250, Cell Signaling, Danvers, MA, USA), rabbit anti-GluA1 (1:1000, Abcam, Cambridge, UK), mouse anti-pERK1/2 (1:200, Sigma–Aldrich, Saint Louis, MI, USA), rabbit anti-pAkt (1:500, Cell Signaling, Danvers, MA, USA), rabbit anti-BDNF (1:250, Abcam, Cambridge, UK), rabbit anti-p11 (1:250, Abcam, Cambridge, UK), goat anti-PSD95 (1:200, Santa Cruz Biotechnology, Paso Robles, CA, USA), mouse anti-β-arrestin 1 (1:100, Santa Cruz Biotechnology, Paso Robles, CA, USA) and rabbit anti-β-arrestin 2 (1:500, Cell Signaling, Danvers, MA, USA). The next day the membranes were washed with Tween 20 at 0.05% in TBS-T and incubated for one hour with conjugated secondary antibodies for fluorescent detection against IgG of mouse or rabbit, at a concentration of 1:15,000, provided by LI-COR Biosciences (Lincoln, NE, USA). The fluorescence signal was detected with an Odyssey CLx Imaging System (LI-COR Biosciences, Lincoln, NE, USA). Blot quantitation was performed by using Image Studio Lite software (LI-COR Biosciences, Lincoln, NE, USA), and densitometry values were normalized with respect to the values obtained with anti-β-tubulin antibody. Results are represented compared to the vehicle group.

### 4.7. Statistics

Data are expressed as mean ± SEM. Differences between two groups were analyzed using a two-tailed Student’s *t*-test. For microdialysis experiments, changes in monoamines and glutamate concentrations were analyzed using repeated measures ANOVA with drug and time as factors, followed by post-hoc Tukey’s multiple comparisons test. The level of significance was set at *p* < 0.05.

## 5. Conclusions

Altogether, our results suggest that elevated transmission of glutamate and do-pamine in the mPFC can underlie the rapid antidepressant-like effects of the 5-HT_1A_ re-ceptor biased agonist, NLX-101. It remains to be determined whether such changes might be shared by other rapid-acting antidepressant drugs. In view of the preferential selectivity of NLX-101 for postsynaptic 5-HT_1A_ receptors localized in the mPFC, it can be argued that its antidepressant-like effects are mediated by 5-HT_1A_ receptors localized to GABAergic interneurons in this brain region. The rapid antidepressant-like effects of NLX-101 are likely mediated by the expression of pERK1/2, pmTOR and p11 and suggest that direct targeting of mPFC 5-HT_1A_ receptors with cortically-biased agonists could be a promising strategy to develop novel and potentially superior antidepressant drugs.

## Data Availability

Data is contained within the article.
